# Testing Students with Special Educational Needs in Large-Scale Assessments – Psychometric Properties of Test Scores and Associations with Test Taking Behavior

**DOI:** 10.3389/fpsyg.2016.00154

**Published:** 2016-02-23

**Authors:** Steffi Pohl, Anna Südkamp, Katinka Hardt, Claus H. Carstensen, Sabine Weinert

**Affiliations:** ^1^Freie Universität BerlinBerlin, Germany; ^2^TU Dortmund UniversityDortmund, Germany; ^3^Humboldt Universität BerlinBerlin, Germany; ^4^Otto-Friedrich-University BambergBamberg, Germany

**Keywords:** special educational needs, competence assessment, large-scale, item response theory, mixture models, test-taking behavior, guessing, missing responses

## Abstract

Assessing competencies of students with special educational needs in learning (SEN-L) poses a challenge for large-scale assessments (LSAs). For students with SEN-L, the available competence tests may fail to yield test scores of high psychometric quality, which are—at the same time—measurement invariant to test scores of general education students. We investigated whether we can identify a subgroup of students with SEN-L, for which measurement invariant competence measures of adequate psychometric quality may be obtained with tests available in LSAs. We furthermore investigated whether differences in test-taking behavior may explain dissatisfying psychometric properties and measurement non-invariance of test scores within LSAs. We relied on person fit indices and mixture distribution models to identify students with SEN-L for whom test scores with satisfactory psychometric properties and measurement invariance may be obtained. We also captured differences in test-taking behavior related to guessing and missing responses. As a result we identified a subgroup of students with SEN-L for whom competence scores of adequate psychometric quality that are measurement invariant to those of general education students were obtained. Concerning test taking behavior, there was a small number of students who unsystematically picked response options. Removing these students from the sample slightly improved item fit. Furthermore, two different patterns of missing responses were identified that explain to some extent problems in the assessments of students with SEN-L.

## Introduction

Large-scale assessments (LSAs) such as the Program for International Student Assessment (PISA; e.g., OECD, 2012), the National Assessment of Educational Progress in the United States (NAEP; e.g., National Center for Education Statistics, 2013), or the German National Educational Panel Study (NEPS; see Blossfeld et al., 2011) generally aim at drawing inferences about competencies and factors influencing competencies and competence development. For this purpose, LSAs usually draw on representative samples of students enrolled in different school forms to cover the whole range of the educational system. In practice, however, most large-scale studies have mainly focused on students in general education. Due to problems of obtaining reliable and valid competence scores, students with special educational needs (SEN) have only been included in the assessments in small numbers and they have often been assessed with reduced item numbers, extended testing time, or otherwise accommodated testing conditions (e.g., Pitoniak and Royer, 2001; Lutkus et al., 2004; Jude and Klieme, 2010). Although considerable effort has been made to find appropriate tests for students with SEN in LSAs, the psychometric quality of the respective competence measures for these students is low. As a consequence, valid conclusions on competencies of students with SEN can hardly be drawn (e.g., Bolt and Ysseldyke, 2008; Lovett, 2010). In the United States, as an example, alternate assessments have recently been designed to assess students with the most significant cognitive disabilities (Nebelsick-Gullett et al., 2015). These facilitate the participation of these students in the assessment and provide information on their level of competencies. However, as these tests do not focus on assessing the competencies of students with and without SEN on the same scale, the respective competence scores of students with SEN may not be directly compared to those obtained from students in general education. International LSAs (e.g., PISA; OECD, 2012), however, do aim to measure competencies of students with and without SEN on the same scale. These studies even go one step further aiming at a comparison of students’ competence levels between countries. A specific challenge in these assessments is that in different countries students with SEN are differently defined and follow different schooling (e.g., schooling within general education or in specific schools). In fact, measuring competencies of students with and without SEN on the same scale is also an important prerequisite for research on the effects of schooling of students with SEN (general education vs. special schools) on students’ competence development (Kocaj et al., 2014).

The present study addresses methodological aspects of the assessment of students with SEN. In the study we focus on students with SEN in learning (SEN-L). Specifically we pursue the question *for which* students with SEN-L we can draw valid inferences on competencies assessed in LSAs. We propose a methodological approach that allows for identifying students with SEN-L for whom measurement invariant test scores with satisfactory psychometric properties were obtained in a LSA. Our approach is applied after data collection, when test administration did not yield satisfactory competence measures. We think that it is worthwhile to identify students with measurement invariant test scores of good psychometric properties at this point. Identifying students with test scores of good psychometric properties allows for drawing valid inferences about competencies of at least some students with SEN-L. Moreover, we are not only interested in identifying those students but also in investigating test-taking behavior (i.e., random guessing and omission or not reaching of items) as one possible explanation for unsatisfactory properties of test scores. This may provide knowledge about the problems students with SEN-L encounter when taking a test. This knowledge, in turn, may inform new strategies of adapting tests to the specific requirements of (subgroups of) students with SEN-L and of formulating test instructions.

### Assessing Competencies of Students with Special Educational Needs

So far, research has shown that the assessment of students with SEN is methodologically challenging. One challenge is to provide tests with appropriate difficulty and item fit. Previous research has shown that test versions designed for students in general education are often too hard for students with SEN, resulting in item misfit and unreliable measures (e.g., Südkamp et al., 2015b). Furthermore, it is a challenge to obtain comparable competence scores for students with and without SEN. Measurement invariance is often considered as a prerequisite for group comparisons (e.g., Lord, 1980; Millsap, 2011). Measurement invariance holds when the item parameters of the measurement model are equal across the respective groups. In Item Response Theory (IRT) models this may be tested by differential item functioning (DIF).

Researchers strongly emphasize the need to evaluate the psychometric properties of a test when testing students with SEN (e.g., Pitoniak and Royer, 2001; Lovett, 2010). So far, results on model fit and measurement invariance of test results are inconsistent. Whereas Lutkus et al. (2004) found good item fit and proved measurement invariance, other researchers (e.g., Koretz, 1997; Bolt and Ysseldyke, 2008; Lovett, 2010) did find indications of strong DIF when comparing item difficulties for students with SEN tested with testing accommodations and for students without SEN tested under standard conditions.

### Testing Students with Special Educational Needs in Learning in the German National Educational Panel Study

The German NEPS is a large-scale longitudinal multi-cohort study that investigates the development of competencies across the lifespan (Blossfeld et al., 2011). Between 2009 and 2012, six representative starting cohorts were sampled, including about 60,000 individuals from early childhood to adulthood. Domain-specific and domain-general competencies of these participants are repeatedly assessed to facilitate investigation of competence acquisition, educational pathways, and returns to education (Weinert et al., 2011). The study aims at providing high-quality, user-friendly data on competence development and educationally relevant processes for the international scientific community. With respect to students with SEN, a series of feasibility studies on these students within this LSA were conducted. These studies aim at answering the question, whether (and how) it is reasonable to include students with SEN in future LSAs.

In these studies the focus is set on a specific group of students with SEN, that is, students with SEN in learning (Heydrich et al., 2013). In our notion, students with SEN-L comprise all students, who are provided with special educational services due to a general learning disability. The group of students with SEN-L composes the largest group of students with SEN in Germany (KMK – Sekretariat der Ständigen Konferenz der Kultusminister der Länder in der Bundesrepublik Deutschland, 2012). In Germany, students are assigned to the SEN-L group when their learning, academic achievement, and/or learning behavior is impaired (KMK – Sekretariat der Ständigen Konferenz der Kultusminister der Länder in der Bundesrepublik Deutschland, 2012) and when students’ cognitive abilities are below normal range (Grünke, 2004). Note that students with specific learning disabilities (e.g., a reading disorder) whose general cognitive abilities are within normal range are not assigned to the group of students with SEN-L. In Germany, the decision of whether a student has SEN in learning is based on diagnostic information as well as on collaborative appraisement by parents, teachers, consultants, and school administrations. About 78% of the SEN-L students in Germany do not attend regular schools but special schools with specific programs and trainings tailored to those who are unable to follow school lessons and subject matter in regular classes (KMK – Sekretariat der Ständigen Konferenz der Kultusminister der Länder in der Bundesrepublik Deutschland, 2012).

In line with previous findings, the feasibility studies conducted within the NEPS showed that it is specifically challenging to obtain measurement invariant (to general education students) competence scores of adequate psychometric quality for students with SEN-L (Südkamp et al., 2015b). We evaluated to what extent the standard test used to assess reading competence of general education students as well as two accommodated test versions are appropriate to yield measurement invariant competence scores with high psychometric quality for students with SEN-L in grade five. The test accommodations included a reduction in test length (i.e., number of items) and a reduction in test difficulty (i.e., difficult items were replaced by items with a lower difficulty).^1^ Results showed that all three reading test versions were suitable for an invariant measurement of reading competence with high psychometric quality in students *without* SEN-L (i.e., in a sample of students without SEN-L in the lowest track of general education). Thus, test accommodations did not threaten comparability of the results in students without SEN-L. For students with SEN-L, the accommodated test versions considerably reduced the number of missing responses and resulted in better psychometric properties than the standard test. Item fit and measurement invariance were, however, not satisfactory enough to ensure a credible assessment. Thus, with the available tests in NEPS it is not possible to obtain comparable scores of reading competence with adequate psychometric quality for students with SEN-L.

### Identifying Students with Special Educational Needs in Learning with Adequate Psychometric Quality of Test Scores

Most of the research dealing with the assessment of students with SEN-L in LSAs has put considerable effort on adapting the tests or the test settings in order to improve the measurement (e.g., Pitoniak and Royer, 2001). Given the constraints on test forms and settings in LSAs, tests may only be accommodated to some extent. In most studies time and expenses for the assessment is restricted. It may be, however, that the test works for a subgroup of students and that unsatisfactory psychometric quality results only for some students with SEN-L. In this study, we follow the idea that students with SEN-L in LSAs might be dividable into a subgroup of students from whom psychometrically acceptable and measurement invariant competence scores have been obtained and a subgroup for which this is not the case. To our knowledge, there is scant research on identifying these two subgroups of students with SEN-L.

### Test-Taking Behavior

There are different possible reasons for unreliable and invalid competence scores in the assessment of students with SEN-L. For example, the test may be too difficult for students with SEN-L, students may lack cognitive skill-level, students may not be used to the test format, or they may not show their full potential in the test (e.g., Heydrich et al., 2013; Südkamp et al., 2015b). All these reasons may manifest themselves in the way students with SEN-L approach the items in the tests, and thus, in their test-taking behavior. Comparing students with learning disabilities to general education students, Scruggs et al. (1985) found that taking a test was less challenging for students without learning disabilities. The authors argued that test-taking skills refer to test-wiseness, which includes time-using strategies, error-avoidance strategies, guessing strategies, and deductive-reasoning strategies. Scruggs and colleagues found that students with learning disabilities show less use of appropriate reasoning strategies than general education students on inferential items. Guessing behavior was only addressed by one questionnaire item, for which the authors did not present specific results. Time-using strategies were not considered in their study.

Most research on test-taking behavior has been performed on students without SEN. Prominent test-taking behaviors considered in previous research are guessing and non-response to questions, as these can be evaluated after data collection without the need to explicitly ask participants about their behavior. Both behaviors can be considered to relate to the aspects of guessing and time-using strategies within the concept of test-wiseness.

Guessing may occur on some or even on all test items, and it can be applied more or less strategically. Being applied strategically, subjects may rather intentionally guess than skip an item, if they don’t know the correct answer and want to optimize their performance (e.g., Dolly and Williams, 1986). This is most prevalent in high-stakes assessments (e.g., Schnipke and Scrams, 1997). In contrast, guessing occurs non-strategically when students unsystematically tick an answer or choose an answer based on other (often superficial) characteristics. For general education students we have learned from qualitative studies (e.g., Nevo, 1989; Anderson et al., 1991) that some students actually guess without any rationale. This may more often occur in low-stakes assessments and may be an indicator of low motivation (Wise and Kong, 2005). It may also be an indicator of problems with the test instruction, item comprehension, or other characteristics of the test. Random guessing is one possible source of aberrant response patterns (e.g., Meijer et al., 1994) and results in problems with item statistics, reliability, and validity (Meijer and Sijtsma, 2001).

Another type of test-taking behavior that often occurs in LSAs is item omission and not reaching the end of the test (e.g., due to timing issues). This behavior results in missing responses. Large numbers of missing responses may lead to problems of test reliability and validity of the estimated ability scores (e.g., Pohl et al., 2014). In that regard, Südkamp et al. (2015b) found that students with SEN-L have a greater number of missing values due to item omission and not reaching the end of the test than low-performing students in general education (i.e., those attending the lowest academic track in general education). These results were found regardless of whether the considered reading test was accommodated to the target group of students with SEN-L or not. Similarly, Koretz (1997) as well as Kato et al. (2007) found that students with SEN were more likely to omit open response items than students without SEN. This finding was mostly stable across grades and several competence domains. Item omission often relates to the difficulty of the item and the ability of the person (e.g., Pohl et al., 2014). When test takers do not know the answer to an item, they are more likely to skip the item as compared to when they do know the answer. This may be especially prevalent for students with SEN-L who are expected to show lower ability than students in general education (e.g., Ysseldyke et al., 1998). Missing values at the end of the test are mainly a result of time constraints when test takers do not manage to finish the test within the given time (Koretz and Barton, 2003).

Within one assessment different persons may use different strategies and certain test-taking behavior may result in low psychometric quality of the test scores. While the test-taking process may well be investigated using qualitative methods, these methods are not feasible for application in large samples. In LSAs, strategy choice cannot be observed directly, but may be inferred from the response patterns of the persons. Some studies that tried to identify different test-taking behavior in tests implemented in LSAs used complex mixture modeling approaches (for an overview see Lau, 2009). These approaches require large data sets as available in the context of LSAs and allow for the identification of unknown groups that are distinguished based on similarities and differences in their response patterns. Mislevy and Verhelst (1990) presented a mixture IRT model that allows identifying random guessing behavior on the whole test. The authors assume that an IRT measurement model holds for the data within one group of students, whereas for a second group unconditional independence of item responses is assumed and the item difficulties are set to a value that describes the chance of a correct response when guessing is applied. These groups are modeled as latent classes and the probability for each test taker to belong to each of the two classes is estimated.

Whereas many studies corroborate the finding that guessing, omission of items, and quitting on the test affect reliability and validity of the test scores of students in general education, no studies exist which draw on these kinds of response behavior to explain low psychometric quality of the tests for students with SEN-L. Gaining insight into the test-taking behavior of students with SEN-L may help us to understand the challenges associated with testing these students.

## Research Objectives

As prior research has shown, assessing competencies of students with SEN-L with tests implemented in LSAs often fails to provide comparable competence scores of adequate psychometric quality. In order to facilitate research on competencies of students with SEN-L, we examined the question *for which students with SEN-L* reliable and comparable competence measures may be obtained with tests available in LSAs. Compared to research on testing accommodations, we shift the focus from *the test* to *the students*. In this study, two major research objectives are addressed: First, we investigate whether it is possible to identify students with SEN-L for whom we may obtain competence measures of high or at least acceptable psychometric quality that are measurement invariant to general education students with tests used in LSAs. Secondly, we investigate how test-taking behavior differs between these two subgroups of students with SEN-L in LSAs and to what extent test-taking behavior may explain low psychometric quality and lack of comparability in LSAs. The types of test-taking behavior we focus on in this study are a) guessing behavior and b) missing responses due to item omission or due to not reaching the end of the test. If our approach proves to be successful, it will not only be possible to draw inferences on competencies of some students with SEN-L in comparison to students in general education—but also to gain knowledge about the problems students with SEN-L encounter when taking tests within LSAs.

## Materials and Methods

### Ethical Statement

*Ethics committee*: The approval of the ethical standards was assured by the National Educational Panel Study. In addition, all tests and questionnaires as well as data collection and data handling procedures were approved by the Federal Ministries of Education in Germany.

*Consent procedure*: The National Educational Panel Study has very high standards for consent procedures. Informed consent was given by the parents as well as the students. The consent procedure was approved by a special data protection and security officer of the National Educational Panel Study.

*Additional ethics details*: The study involved students with special educational needs in learning. The Federal Ministries of Education in Germany and the data security officer of the National Educational Panel Study approved the study. Informed consent was given by parents, students, and educational institutions to take part in the study. Students (as well as all other parties) could abort their participation at any time in the study. Test administrators received intensive and specific training. The assessments were conducted in a motivating child-oriented manner.

### Sample and Design

For this study we used data from two different studies of students in grade nine that were conducted in 2011 within the NEPS. These studies comprise (a) a representative sample of general education students (main sample) and (b) a sample of students with SEN-L. In the main sample there were 13,933 general education students (for more information on the NEPS main samples see Aßmann et al., 2011). All subject or their legal guardians gave written informed consent according to the laws of the German federal states. Thirty six of these students had fewer than three valid responses and were excluded from the analyses, resulting in a total number of *N* = 13,897 students that entered the analyses. On average, these students were *M* = 15.72 (*SD* = 0.64) years old and 49.8% were female. The sample of students with SEN-L draws on a feasibility study with 403 students who were exclusively recruited from special schools for children with SEN-L in Germany. Two students had fewer than three valid responses and were excluded from the analyses, resulting in *N* = 401 students considered in the analyses. Students in this sample were on average *M* = 16.00 (*SD* = 0.64) years old and 43.9% were female. Students participated in the study voluntarily, so student and parental consent was necessary.

### Measures and Procedures

Within both samples, reading as well as mathematical competence were assessed. In this study, we focus on the assessment of reading competence. Within the NEPS, the reading competence assessment focuses on text comprehension (Gehrer et al., 2013). Individuals were presented five texts of different text types, and they were expected to respond to questions regarding the content of these texts. These questions featured different response formats including multiple choice (MC) items, complex MC tasks, and matching tasks (see Gehrer et al., 2012). MC items comprise four response options with one of them being the correct response. Complex multiple choice (CMC) tasks present a common stimulus that is followed by a number of MC items with two response options each (asking for agreement or disagreement with a given statement). The common stimulus of matching (MA) tasks requires the assignment of a list of response options to a given number of statements (e.g., headings need to be assigned to text paragraphs). Thereby, the number of items within the MA task reflects the number of statements. Students had 30 minutes to complete the test. Our analyses were based on a standard reading test designed for general education students as well as on an accommodated test version, which was reduced in the number of items (called “reduced reading test”). The reduced test consists of the same items as the standard test except for the last text and its respective seven items plus an additional three difficult items. As such, the reduced test contains fewer items than the standard test. For testing general education students, the standard reading test proved to have good psychometric properties (Haberkorn et al., 2012).

Students in general education took the standard reading test. Students with SEN-L took the standard reading test (*n* = 204) or the reduced reading test (*n* = 199) by random assignment. To facilitate a stable estimation of our models, we did not differentiate between the two test versions used for students with SEN-L but analyzed the two groups together. This ensures a sufficient sample size for the estimation. Although this ignores possible differences between the two test versions, we do not expect these to systematically change our results. With the exception of one item, item difficulty parameters did not substantially differ between the two test versions (see Table A1 in the Supplementary Material). Low psychometric quality of the measurement and measurement non-invariance to general education students occurred on both test versions and possible differences in test-taking behavior between the two test versions can be evaluated in the analysis. In order to evaluate the impact of the test version on test scores, we report on differences in the results of the analyses between the two test versions.

For students with SEN-L a large number of missing responses occurred on the last text of the standard test; in the reduced test version, the entire text was missing as part of the test accommodation. Since only few valid responses remained, we excluded the corresponding seven items of the last text in the standard test from the analyses. Thus, our analyses are based on 32 items of the standard test and 29 items of the reduced test. There were 20 and 17 MC items, 8 and 8 items referring to 3 CMC tasks, and 4 and 4 items referring to 1 MA task in the standard and in the reduced test, respectively. Regarding the position, there was no systematic order of items by their response format (see Table A1 in the Supplementary Material for the item order). The only MA task included in the two reading test versions referred to the first text. CMC tasks as well as simple MC items were distributed over the whole test. Note that in order to also evaluate the fit of all subtasks of CMC and MA tasks, all items regardless of their response format were treated as single dichotomous items in the analyses.^2^

### Analyses

In order to evaluate the quality of measurement, we first applied a Rasch model to the whole sample of students with SEN-L. With this analysis we gained information on the psychometric properties and measurement invariance of the competence measures of the whole sample of students with SEN-L. These results also served as benchmarks; we aimed at improving these indices in subgroups distinguished based on a subsequent mixture modeling approach.

#### Scaling the Data

We scaled the data in accordance with the scaling procedure for competence data in the NEPS (Pohl and Carstensen, 2012, 2013) using a Rasch model (Rasch, 1960). We fitted the Rasch model using both ConQuest 2.0 (Wu et al., 2007) and M*plus* Version 6.1 (Muthén and Muthén, 2010b). ConQuest was used since it provides a number of different fit indices. Estimation in M*plus* was carried out in order to allow for comparison to the mixture models described later. As shown by Muthén and Muthén (2010a), the parameter estimates from ConQuest and M*plus* are mutually transferable. According to the notation of latent factor analysis used in the General Latent Variable Modeling Framework of M*plus*, the probability of a correct response to item *i* given a certain ability can be described as

$$P(Y_{i}=1|\eta )=\frac{1}{1+\exp-\left(-\tau_{i}+\eta \right)}.$$

Therein, *η* denotes the latent ability of the subjects and the thresholds *τ_i_* indicate the estimated item parameters. Using marginal maximum likelihood estimation, missing responses were ignored in the parameter estimation (for the rationale see Pohl et al., 2014).

#### Identifying Students with SEN-L with Comparable Test Scores of Appropriate Psychometric Quality

In order to evaluate psychometric quality and comparability of persons’ competence measures, the fit of response patterns of the students with SEN-L to given item parameters from the measurement model for general education students was investigated using mean square error statistics for persons (Wright and Masters, 1990). These person fit statistics were obtained using the software ConstructMap, Version 4.6 (Kennedy et al., 2010). The software relies on ConQuest for estimation procedures. Although no clear guidelines for judging the size of person fit statistics exist, Linacre (2002) suggested to evaluate person fit statistics between 0.5 and 1.5 as productive for measurement, between 1.5 and 2 as being “unproductive,” and values greater than 2 as distorting the measurement system due to unexpectedly large randomness inherent in the responses. Values below 0.5 are less productive for measurement, but not degrading. In order to evaluate the fit of the response patterns of a student with SEN-L to the measurement model obtained for the sample of general education students, we calculated the unweighted person fit (outfit) using item difficulty parameters estimated for the general education students. In this way, the person fit statistic provides information on how well response patterns of students with SEN-L conform to the measurement model valid for general education students. Thus, it is both, a measure of fit to the measurement model and a measure of comparability to general education students.

#### Identifying Inter-individual Differences in Test-Taking Behavior

In order to identify inter-individual differences in test-taking behavior, we applied two mixture models to the data. In the first model we explicitly modeled random guessing for a subgroup of test takers. In the second model we captured differences in testing-taking behavior based on the missing values in the test.

##### Guessing

In the first mixture model we tried to identify students in the sample who unsystematically responded to the questions, regardless of the content. We also refer to these students as *random guessers*. For this purpose, we specified a mixture distribution Rasch model (Rost, 1990) with two latent classes. The respective model equation written in the General Latent Variable Modeling Framework used in M*plus* is

$$P(Y_{i}=1|g,\;\eta_{g})=\frac{1}{1+\exp-\left(-\tau_{ig}+\eta_{g}\right)},$$

with *g* indicating the latent class. We imposed restrictions on this model. We assumed that in the first class the IRT model equals the Rasch model (see Eq. 1). For the class comprising the random guessers we assumed that the probability of answering correctly does not depend on ability but equals the chance of a correct response. This chance is calculated as one over the number of response options. Since the items in the reading test have different numbers of response options, different item thresholds corresponding to the different response probabilities are used. Computing the response probabilities as described above, results in the threshold parameters *τ_i_* to be fixed at 1.0986, 0, and 1.6094 for MC items, items within CMC tasks, and items within the MA task, respectively.

Thus, the model in the second class is described by

$$P\left(Y_{i}=1\right)=\frac{1}{1+\exp-\left(-\tau_{i}\right)}\,$$

with threshold parameters fixed to the values described above. Note that the response probability in this class is only determined by the item (i.e., the item format) but not by the person ability. The mean and variance of the ability variable in the second class were set to zero. For reasons of identification, the sum of the probabilities for each person to belong to each of the two classes is fixed to one. Note that this model is closely related to the pure guessing model of Mislevy and Verhelst (1990). However, in contrast to our approach, Mislevy and Verhelst use this model for identifying non-motivated students aiming at unbiased estimates of the model parameters.

We evaluated the fit of the model by comparing Akaike Information Criterion (AIC; Akaike, 1974) and the Bayesian Information Criterion (BIC; Schwarz, 1978) indices to those of the one-group Rasch Model (see Eq. 1). The entropy^3^ criterion (Ramaswamy et al., 1993) as well as the average posterior probabilities per class served to judge the classification quality. Finally, in subsequent analyses we investigated the improvement of item fit and comparability for a subgroup of students with SEN-L without the random guessers.

##### Missing Responses

A second indicator of test-taking behavior was derived from special kinds of missing responses present in the competence tests. As such, missing values due to omitting items (i.e., items not responded to within the test; items are coded as omitted when there is at least one valid response on following items) and missing values due to not reaching the end of the test within the given time limit (i.e., all items not responded to after the last valid response) were considered in this analysis. Since omission was found to be multidimensional with the response formats describing three dimensions of the omission propensity, we considered three different measures of item omission: omission of MC [O_MC_] items, omission of items within CMC tasks [O_CMC_], and omission of items within the MA [O_MA_] task. We used three manifest variables, which were computed for each person as the percentage of item omissions in the respective response format relative to the total number of items of that response format.^4^ As suggested by Rose et al. (2015), a single indicator [NR] referring to the percentage of not reached items relative to the total number of presented items was used in the analyses. In our analyses we used the four missing indicators; no measurement model of the item responses or the item responses themselves were used. We supposed different test-taking behavior with respect to how many items were omitted or not reached. However, we had no differential hypotheses on how the students would differ in their behavior. We therefore specified a mixture model including all four missing indicators (three for item omission and one for not reached items). Due to the small sample size we limited the number of classes to two. We allowed all parameters estimated in each class, that is means, variances, and covariances of the missing indicators, to vary across classes. We evaluated classification quality (entropy as well as average posterior probability per class) and investigated the pattern of missing responses based on the estimated means, variances, and correlations in the two classes. In further analyses we investigated whether the two classes differed with regard to item fit and measurement invariance.

#### Measures of Item Functioning

In order to investigate the measurement quality of the reading test for students with SEN-L, we evaluated different item fit measures (as indicators of the psychometric quality of the test) as well as measurement invariance (as indicator of comparability).

##### Item Fit

Item fit measures included the weighted mean square (WMNSQ; Wright and Masters, 1990), item discrimination, point biserial correlation of the distractors with the total score, and the empirically approximated item characteristic curve (ICC) for the item. We evaluated WMNSQ values greater than 1.15, item discriminations below 0.2, and correlations of distractor categories (that are chosen by at least *n* = 20 subjects) with the total scores greater than 0.05 as noticeable indications of misfit^5^. All of the fit measures provide information on how well the items fit a unidimensional Rasch model. As shown, fit statistics depend on the sample size; the larger the sample size, the smaller the WMNSQ and the greater the *t*-value. Discrimination, on the other hand, depends on the distribution of the scores. If the variance of the responses to an item is small (because the item is too easy or too difficult), its discrimination will be low. Since the subgroups of students with SEN-L that will be separately analyzed differ in sample size, we considered all of the evaluation criteria described above.

##### Measurement Invariance

We tested for measurement invariance by means of DIF analyses comparing the estimated item difficulties in the sample of students with SEN-L to the estimated item difficulties of the same items for students in the main sample of the NEPS. We estimated DIF in a multi-facet Rasch model (cf. Linacre, 1994), in which the response probabilities were modeled as a function of ability level, item difficulty, sample (general education or SEN-L), and item by sample interaction. For identification and comparison, the mean of the item difficulties were set to zero in both samples. Means of the latent ability were freely estimated within each group. In line with the benchmarks chosen in the NEPS (Pohl and Carstensen, 2012, 2013), we considered absolute differences in item difficulties greater than 0.6 logits to be noticeable and absolute differences greater than 1 logits to be strong DIF.

Note that with this analysis we cannot distinguish between DIF and item impact. If there was a systematic advantage for students in general education as compared to students with SEN-L on all items, we would not detect this with the present DIF-analyses but this would contribute to the difference in the means of the latent ability. In any case, the absence of DIF is a necessary (but not sufficient) prerequisite for the different measurements to be on the same scale. This is what we evaluate with our analyses.

#### Associations Between Test-Taking Behavior and the Psychometric Properties and Comparability of Students’ Test Scores

We checked whether a good model fit and invariant competence measures might be obtained for certain subgroups that are characterized by different test-taking behavior. Using measures of item functioning, we first evaluated whether the test produced measurement invariant competence scores with adequate psychometric properties for the respective subgroup. For this purpose, we evaluated item fit and measurement invariance (as described above) for the subgroups characterized by the different test-taking behaviors. We furthermore checked whether the classification of persons to a certain test-taking behavior (guessing or missing pattern) corresponds to person fit. If test-taking behavior explains to some extent the low psychometric properties of the test, then there should be a relationship between test-taking behavior and person fit indices.

## Results

As a benchmark, we will first present the results on item fit and measurement invariance for the whole sample of students with SEN-L. We will then identify students for whom test scores with appropriate psychometric quality, that are at the same time measurement invariant to students in general education, can be obtained. Next we will identify differences in test-taking behavior between students with SEN-L. Finally, we will evaluate whether differences in test-taking behavior may explain the quality of competence measurement.

### Test Quality in the Whole Sample of Students with SEN-L

**FIGURE 1:**
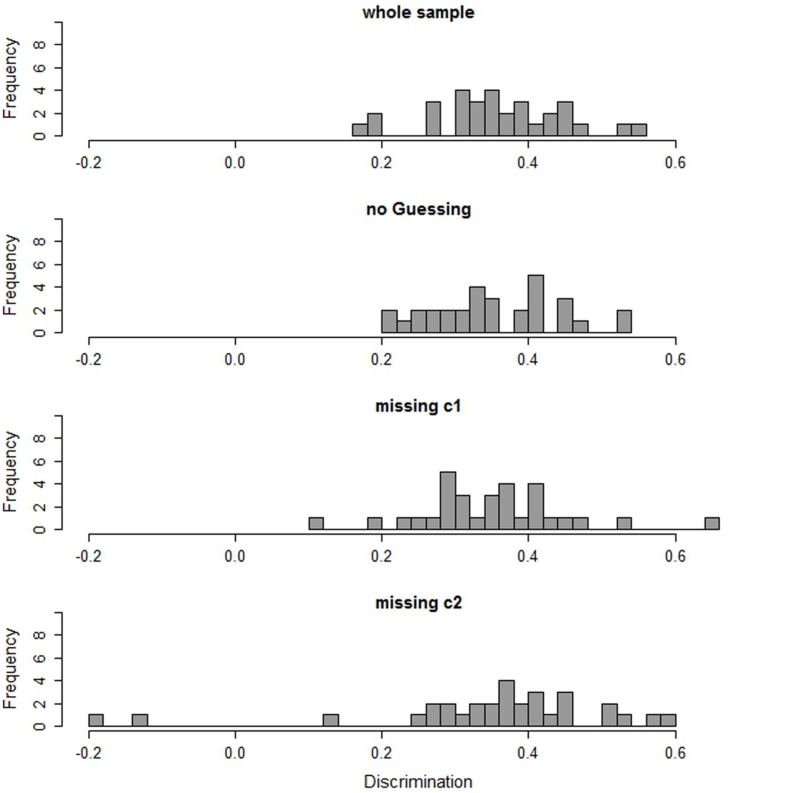
**Item discriminations in the whole sample, in the subgroup of non-guessers (no guessing), and in the two subgroups with different missing patterns (missing c1 and missing c2)**.

First, we evaluated item fit for the whole sample of students with SEN-L. Most of the items show satisfactory fit indices (see **Figure 1**). The average item discrimination for students with SEN-L is 0.36. Two items show a discrimination below 0.2. There is a tendency that item discrimination is lower for difficult items than for easy items (*r* = -0.34)^6^. Evaluation of further fit measures confirms these results. Considering WMNSQ, ICC, and distractor analyses, there are six items that show indications of misfit on at least one of these indices (detailed information on fit indices can be found in Table A1 in the Supplementary Material).

**FIGURE 2:**
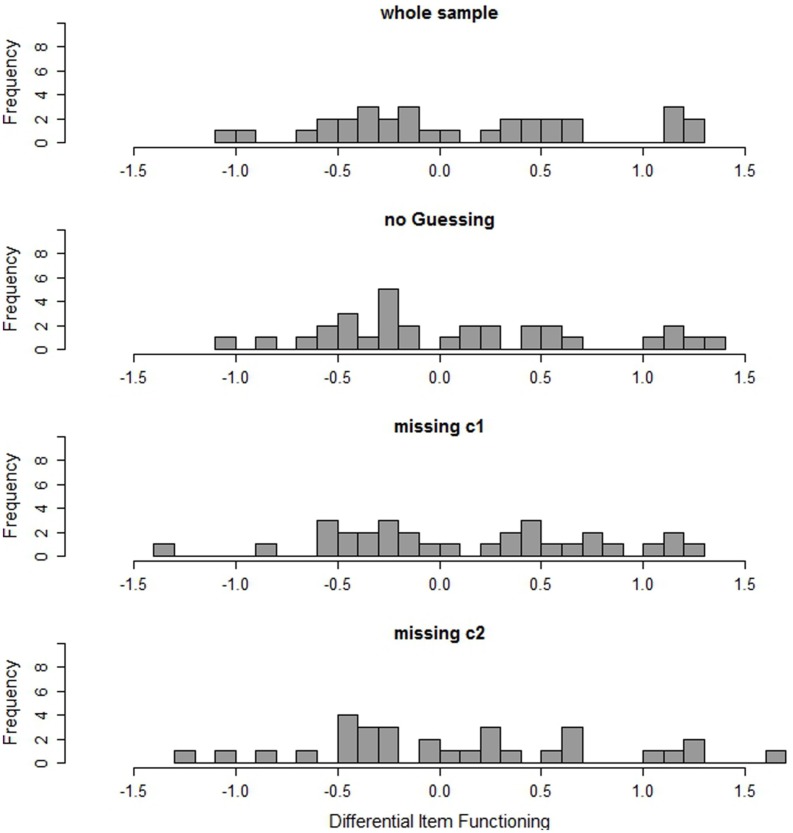
**DIF (in logits) of the items in the whole sample, in the subgroup of non-guessers (no guessing), and in the two subgroups with different missing patterns (missing c1 and missing c2)**.

Considering measurement invariance of the test, ten of the 32 items show differences larger than 0.6 logits in the estimated item difficulties between students with SEN-L and general education students (**Figure 2**); six of these ten items even have absolute DIF greater than 1 logit. These results provide evidence that the reading test may be measuring a different construct for students with SEN-L than for general education students. Summarizing these results, the psychometric properties of the test assessing students with SEN-L may not be sufficient. The results indicate that although the test may allow for a measurement of competencies of general education students, it does not necessarily result in psychometrically appropriate and, in particular, invariant measures for students with SEN-L.

### Identifying Students with SEN-L with Comparable Test Scores of appropriate Psychometric Quality

Persons whose responses do show a good model fit and whose competence assessment is not measurement-invariant to general education students were identified using person fit statistics. Person outfit statistics range from 0.09 to 4.27 with a mean of 1.16 and a standard deviation of 0.51. Seventy-seven persons (19.2%) have a person outfit value greater than 1.5 and 20 students (5%) have a person outfit value greater than 2. There were no significant mean differences of the person outfit with regard to the test version the students were administered [Δ_outfit_ = 0.078, *t*(399) = 1.538, *p* = 0.125, *d* = 0.154], nor was there a relation between whether persons were flagged as having aberrant response patterns (i.e., outfit > 1.5) and the test version they were administered (Φ = -0.066, *p* = 0.186). These results show that for both test versions there is a considerable number of students for whom an invariant measurement of adequate psychometric quality may be achieved. Note, that a clear-cut identification is difficult as the evaluation of the person fit indices relies on benchmarks which are to some extent arbitrary.

### Identification of Inter-Individual Differences in Test-Taking Behavior

In this section we present the results on identifying subgroups of students who show different forms of test-taking behavior. We will first report the results on guessing and then on different patterns of missing responses.

#### Guessing

The model fit of the guessing model (logLikelihood = -5656, number of parameters = 34, AIC = 11380.16, BIC = 11515.95) is better than that of the one-group Rasch model (logLikelihood = -5663, number of parameters = 33, AIC = 11393.271, BIC = 11525.072). The classification quality is satisfactory (entropy = 0.83). The average model-based posterior probability for the class of random guessers was 0.71, whereas non-guessers were assigned to the other latent class with an average posterior probability of 0.96. Of the 401 students, only 5% were assigned to the guessing class. The classification to the classes is independent of the test version (Φ = 0.020, *p* = 0.687). Although this number seems to be small, it is not negligible.

#### Missing Responses

Two classes were postulated in the missing model. One-hundred-eighty-six (46%) of the students were assigned to the first class and 215 (54%) were assigned to the second class. The classification accuracy was high (entropy = 0.91, with average posterior probabilities of 0.97 and >0.99, respectively). The students could be classified very clearly into one of the two classes. Note that since the model is saturated within each class and since there is no model for comparison, no model fit is presented.

The two classes differ in the number of missing responses as well as in the pattern of missing responses as indicated by response format and missing type. As can be seen in **Table 1**, the first class is characterized by overall small rates of missing values, whereas the second class shows rather great numbers of missing values. This is true for both types of missing responses. Only for MC items, the second class shows considerably lower omission rates than the first class. Class members of the second class more likely omit items of complex response formats than do class members of the first class. Furthermore, all correlations between the different missing indicators differ considerably between the classes (see **Table 2**). In the first class, the number of missing responses correlates medium to high across all missing types and response formats. Students who tend to omit items of a certain response format also tend to omit items of other response formats and do not reach many items. This is different in the second class. Here, omission does not occur coherently across response formats, but correlates rather low. Furthermore, there are negative correlations between the number of not reached items and the number of omitted items. This might be due to the fact that the number of omitted and the number of not reached items are dependent and a large number of not reached items automatically reduces the maximum number of items one may omit. Latent class assignment was slightly related to the test version (Φ = 0.217, *p* < 0.001). The number of students receiving the standard test that were assigned to the second missing class was higher (64.40%) than the number of students receiving the reduced test (42.71%). Summarizing the results, it seems that the first class shows a rather consistent response behavior with a low number of missing values, whereas students in the second class skipped complex items and generally did not finish the test. Consistent with previous findings, the reduced test version resulted in fewer missing responses. These were more consistent across types of missing responses than for the standard test.

**Table 1:** Estimated means and variances (standard errors) of the missing response indicators for the relative number of not reached items (NR), omitted multiple choice items (O_MC_), omitted complex multiple choice items (O_CMC_), and omitted matching items (O_MA_) in the first and in the second class of the missing model.

	c1 (*n* = 186)		c2 (*n* = 215)	
		
	Mean	Variance	Mean	Variance
NR	1.29 (0.672)	23.757 (12.257)	27.30 (1.401)	447.490 (40.472)
O_MC_	3.53 (0.842)	109.483 (38.366)	1.25 (0.281)	10.335 (2.912)
O_CMC_	2.72 (0.817)	109.818 (58.397)	8.49 (1.138)	270.828 (36.303)
O_MA_	4.338 (1.433)	366.199 (124.906)	19.94 (2.561)	1383.823 (147.031)


**Table 2:** Estimated correlations (standard errors) between missing response indicators for the relative number of not reached items (NR), omitted multiple choice items (O_MC_), omitted complex multiple choice items (O_CMC_), and omitted matching items (O_MA_) in the first and in the second class of the missing model.

	c1 (*n* = 186)	c2 (*n* = 215)
cor(NR,O_MC_)	0.454 (0.126)	-0.225 (0.052)
cor(NR,O_CMC_)	0.207 (0.176)	-0.473 (0.043)
cor(NR,O_MA_)	0.634 (0.183)	-0.295 (0.068)
cor(O_MC_,O_CMC_)	0.724 (0.100)	0.310 (0.091)
cor(O_MC_,O_MA_)	0.746 (0.067)	0.357 (0.088)
cor(O_CMC_,O_MA_)	0.493 (0.120)	0.085 (0.068)


### Associations Between Test-Taking Behavior and the Psychometric Properties and Comparability of Students’ Test Scores

After having identified subgroups with different test-taking behavior, we tested whether this behavior explains the low psychometric properties and the measurement non-invariance of the test in the whole sample of students with SEN-L.

#### Guessing

Since the sample of students who were classified as random guessers is too small for a separate scaling, only the results of the non-guessing class are presented and compared to the scaling results of the whole sample of students with SEN-L. The second histogram in **Figure 1** shows the discriminations of the items in the non-guessing class. To facilitate comparison, the discriminations estimated in the whole sample are depicted at the top of the figure. **Figure 1** illustrates that there are no considerable differences in the discriminations. In fact, the average discrimination in the sample of non-guessers is the same (0.36) as for the whole sample. Also, the number of items being flagged by the WMNSQ is the same in both samples. However, no item in the sample of non-guessers exhibits suspicious discriminations, whereas in the whole sample two items had noticeable discriminations below 0.2. Furthermore, in the sample of non-guessers, only four items (instead of five, as in the whole sample of students with SEN-L) showed inconsistent point-biserial correlations of distractor-choice with the total score. Regarding measurement invariance (see **Figure 2**), there is no improvement for the sample of non-guessers as compared to the whole sample of students with SEN-L. The average absolute difference in item difficulties is 0.54 logits for the whole sample and 0.52 logits for non-guessers. More detailed information on item fit and DIF can be found in Table A2 in the Supplementary Material.

Second, we investigated whether classification into the guessing class explains low psychometric quality and non-invariance of the measurement for some of the persons. Person fit statistics of the random guessers clearly indicate the misfit of their response patterns. Eighteen (81.8%) of the 22 students identified as random guessers were also flagged by the person fit statistics and eight (36.4%) of them even had outfit values greater than 2. The person fit values for the non-guessing class ranged from 0.09 to 4.17 with a mean of 1.11 and a standard deviation of 0.46. Fifty-nine (15.6%) students had outfit values greater than 1.5 and 12 (3.2%) greater than 2. Person fit statistics indicate that, after excluding students assigned to the class of random guessers, the response patterns of the remaining students better conform to parameters estimated for general education students than the response patterns of the whole sample of students with SEN-L. Thus, random guessing of some students is associated to some extent with the low psychometric quality of test results in the group of students with SEN-L.

#### Missing Responses

The two groups of students show distinct item fit measures. In the first class there is a slight improvement in fit measures as compared to the whole sample of students with SEN-L, whereas in the second class measurement is considerably worse (**Figure 1**). Although the mean of the discriminations does not differ between the groups and the whole sample of students with SEN-L and neither does the number of items with inconsistent distractor correlations, the number of items with insufficient fit values on the other indices does differ. In the first class only one item shows a low discrimination and a low WMNSQ. On the contrary, in the second class four items have a discrimination below 0.2 and two of these even have discriminations below zero. Regarding WMNSQ values, two items show values greater than 1.15 (these are even greater than 1.3) in the second class, whereas it is just one item with a WMNSQ of 1.18 in the first class. Concerning measurement invariance, there are differences between the groups (**Figure 2**). In both classes there are five items with a considerable DIF being greater than 0.6 (but less than 1) logit. However, while in the first class there are five items with DIF being greater than one, there are seven in the second class. The largest DIF was 1.65 logits in the second class. It is 1.30 in the first class and 1.27 logits in the whole sample. More detailed information on item fit and DIF can be found in Tables A3 and A4 in the Supplementary Material.

There is some relation between missing patterns and the psychometric quality of test scores. Person outfit values are slightly better in the first (*M* = 1.16, *SD* = 0.40, ranging from 0.09 to 2.52) than in the second missing class (*M* = 1.16, *SD* = 0.59, ranging from 0.09 to 4.17). The variance of the person fit differs significantly between the two groups [*F*(1,399) = 9.98, *p* = 0.002]. Aberrant response patterns as indicated by person outfit values greater than 1.5 (greater than 2) are found for 18% (4%) of the persons in the first missing class and 21% (6%) in the second missing class. The results show that the missing classes explain the low psychometric quality in the whole sample to some extent. The item fit indices and measurement invariance of the competence measures is slightly lower in the subgroup of students who show high and inconsistent missing patterns.

## Discussion

Assessing students with SEN-L in LSAs is challenging. However, we showed that it is possible to identify a subgroup of students with SEN-L who yield test scores of adequate psychometric quality and comparability. Credible competence scores may not be obtained for all but for some students with SEN-L within LSAs. Identifying those students enables researchers to investigate competence development and influencing factors for at least some students with SEN-L and to directly compare competence measures of students with SEN-L to those obtained from general education students. In this study, we focused on students with SEN in learning. Our methodological approach can also be applied to studies that aim at assessing even more heterogeneous samples of students with SEN.

Although our approach allows to draw inferences on competencies of students with SEN-L, even when test scores of the whole sample are not of satisfactory quality, the proposed methodology also has its limitations. Students with test scores of appropriate quality are a systematically selected subgroup of students with SEN-L and are by far not representative of that group. Thus, results on competencies in this specific group cannot easily be generalized to all students with SEN-L. In order to draw more general conclusions, the difference between students with test results of adequate psychometric quality and those without needs to be investigated and described. In our study many background variables (cf. NEPS, 2013) are available for all students with SEN-L; these may be used to statistically adjust for this selection. Note that in international LSAs like PISA a selection of students with SEN, to be included in the assessment, is made *a priori* to the data collection; however, this selection is not based on objective criteria but “intellectually disabled students (…) who are emotionally or mentally unable to follow even the general instructions of the assessment” may be excluded from the assessment based on the judgments of school principals or school coordinators (OECD, 2012, p. 59). Thus, a selection is already present in the available data on competencies of students with SEN in LSAs. Drawing, however, on background data—as in our approach—we can at least describe the selection and try to account for it (or to describe the target population for which conclusions can be drawn).

In our study, we identified differences in test-taking behavior that are associated with the psychometric quality of test scores of students with SEN-L. This provides valuable information for future assessments of students with SEN-L in LSAs. Unsystematically picking response options throughout the entire test seems to be a minor problem. The results on missing responses indicate that some students with SEN-L may have problems with more complex response formats. This is corroborated by the fact that DIF in the whole sample is to some extent related to item format, with items of MA format being specifically more difficult for students with SEN-L than for students in general education. Items of complex response format may in further studies either be removed or better instructed. However, the considered test-taking behavior does not fully explain differences in the psychometric quality of test scores. Interestingly, DIF between students with SEN-L and general educational students was not related to item difficulty; instead, for students with SEN-L especially the items at the beginning of the test were more difficult than for students in general education (correlation between position of the item in the test and DIF being *r* = -0.58). This may indicate that students with SEN-L need some time to get used to the testing procedure. It may be worthwhile for further assessments to include more exercises on test-taking in the test instruction.

There are also some limitations to our study. We only focused on one competence domain (reading) in one grade level (grade nine). Validation of our results across different age groups and competence domains would be desirable. In previous research we could corroborate the findings of the present study on non-satisfactory item fit and DIF for assessing reading competence in a sample of grade five students with SEN-L (Südkamp et al., 2015b) as well as for the assessment of mathematical competencies of grade nine students with SEN-L (Südkamp et al., 2015a). Investigating whether similar test-taking behavior may be found in these studies as well would be a worthwhile endeavor to cross-validate the present findings.

Within this study only a limited number of types of test-taking behavior could be taken into account. We considered, for example, only guessing throughout the whole test. It is hard to model but plausible to assume that students guess only on some items. Although previous research (e.g., Scruggs et al., 1985) has shown that students with SEN are more likely to apply guessing non-strategically, our approach does not allow us to distinguish strategic and non-strategic guessing behavior. Another test-taking behavior may be picking response options not based on the content of the text, but based on additional features (such as general knowledge). We are also aware that with *post hoc* analyses of data from LSAs, we could not directly assess test-taking behavior. Aberrant response and missing patterns are only indicators of test-taking behavior. Other strands of research combine competence measures with explicitly assessed effort measures to approach unmotivated test-taking behavior (Zerpa et al., 2011) or rely on response times as implicit measures for the same purpose (e.g., Wise and Kong, 2005). An integration of different approaches and research traditions might be valuable to gain deeper insights into the relationship between psychometric quality of test scores and test-taking behavior of students with SEN-L. In order to validly draw conclusions on the test-taking behavior of test takers in LSAs, more specific studies in addition to LSAs are necessary. However, as LSAs are important for gaining knowledge on a large sample of students with SEN-L and as problems with test scores of dissatisfactory psychometric quality occur therein, this research may help to close the gap between small, informative, qualitative studies and the powerful LSA studies. In further studies, it may also be worthwhile to compare the test-taking behavior of students with SEN-L to those in general education.

The present study provides a new way of approaching data analysis of students with SEN-L in LSAs. While previous studies mainly focused on accommodating tests—quite often with the drawback that test results were no longer comparable to general education students receiving no accommodations—this study focused on the prospects of available tests and testing conditions in LSAs in order to assess competencies in students with SEN-L. Instead of modifying the test, we introduced a methodological approach that allows for identifying students for whom tests used in LSAs produce comparable competence measures of adequate psychometric quality. Our study may also stimulate further research aiming at understanding test taking problems in students with SEN-L. Our research may also help to find evidence-based criteria for the inclusion or exclusion of students with SEN-L from LSAs. Furthermore, our results may provide a basis for new strategies of adapting tests to the specific requirements of (subgroups of) students with SEN-L and of giving test instructions and, thus, help to move forward the inclusion of students with SEN in LSAs.

## Author Contributions

All authors listed, have made substantial, direct and intellectual contribution to the work, and approved it for publication.

## Conflict of Interest Statement

The authors declare that the research was conducted in the absence of any commercial or financial relationships that could be construed as a potential conflict of interest.

## Footnotes

1 Note that test accommodation is only possible to some extent. For linking test scores of students with SEN-L to those of general education students within a nonequivalent-group anchor item design, a sufficient number of common items is necessary.

2 As such, we did not account for possible local dependencies between items referring to the same task. Note that in the final scaling model in NEPS, in order to account for these dependencies, the responses to CMC and MA items are aggregated to polytomous variables indicating the number of correctly solved subtasks (e.g., Ferrara et al., 1999).

3 The entropy is a standardized summary measure which reflects the accuracy of classification of subjects based on their posterior class probabilities. Values range from 0 to 1, with 1 indicating a very clear delineation of classes.

4 There are models (cf. Holman and Glas, 2005) that depict the omission propensity as a latent variable indicated by the omissions on the individual items. We refrained from using latent measures of the missing propensity since this would pose severe estimation problems for the given sample size.

5 The benchmarks are in line with the benchmarks used in NEPS (Pohl and Carstensen, 2013). Note, that these are not strict benchmarks. We choose these values for describing the results of our analyses.

6 For computation of the correlation we used item difficulty estimates from the sample of general educational students, as these estimates are valid.
