# Supplementary material for: Testing Students with Special Educational Needs in Large-Scale Assessments – Psychometric Properties of Test Scores and Associations with Test Taking Behavior
Source: Front Psychol. 2016 Feb 23;7:154. doi: 10.3389/fpsyg.2016.00154 (PMC4763047; doi:10.3389/fpsyg.2016.00154)
Supplement: Supplementary file 1 [file Table_1.PDF]

Supplementary Material

Appendix A: Detailed results of scaling

Table A1
Results of scaling all students with special educational needs in learning (both test versions).

Item	Item Format	Text	DIF standard vs reduced[1]	Difficulty	WMNSQ	t-value (WMNSQ)	Discrimi-nation	DIF SEN-L vs gen-ed[2]
1	MC	1	-0.118	-1.442	1.02	0.3	0.31	0.416
2	MC	1	0.090	-2.16	0.98	-0.2	0.33	0.498
3	MC	1	-0.242	0.553	1.00	0	0.39	0.382
4	MA	1	0.050	0.441	0.98	-0.5	0.44	1.11
5	MA	1	0.122	1.13	0.89	-1.9	0.54	1.254
6	MA	1	-0.094	1.48	0.97	-0.3	0.38	1.154
7	MA	1	0.612	1.501	1.00	0	0.32	0.074
8	CMC	1	0.232	-1.855	0.99	-0.1	0.34	-0.058
9	CMC	1	0.118	-1.205	1.03	0.5	0.35	-0.314
10	MC	2	-0.028	0.182	0.95	-1.2	0.46	0.652
11	MC	2	0.428	0.919	1.01	0.1	0.37	-0.274
12	MC	2	-0.368	-0.035	0.96	-1.1	0.46	0.508
13	MC	2	0.486	0.701	1.09	1.9	0.27	1.274
14	MC	2		1.44	1.09	0.8	0.27	-0.996
15	MC	3	-0.012	0.17	1.03	0.7	0.20	0.606
16	MC	3	-0.234	-0.128	0.90	-2.6	0.39	1.138
17	CMC	3	0.492	-1.619	0.92	-0.9	0.55	0.24
18	CMC	3	-0.266	-2.023	0.95	-0.5	0.46	-0.252
19	CMC	3	-0.116	-0.968	0.93	-1.4	0.36	-0.116
20	CMC	3	-0.178	-1.432	0.96	-0.5	0.48	-0.622
21	MC	3	0.068	-0.33	1.00	-0.1	0.40	0.34
22	MC	3	0.112	-0.378	0.99	-0.3	0.43	0.516
23	MC	3	-0.284	-0.63	1.02	0.5	0.41	-1.048
24	MC	3	-0.348	0.542	1.08	1.7	0.34	-0.516
25	MC	4	0.260	-0.898	1.06	1.2	0.28	-0.492
26	MC	4	-0.030	0.395	1.05	1	0.35	-0.108

Item	Item Format	Text	DIF standard vs reduced[1]	Difficulty	WMNSQ	t-value (WMNSQ)	Discrimi-nation	DIF SEN-L vs gen-ed[2]
27	MC	4		0.604	1.11	1.3	0.19	-0.34
28	MC	4	-0.106	0.017	1.04	0.8	0.36	-0.188
29	MC	4	0.034	-0.667	1.03	0.6	0.32	-0.518
30	MC	4		0.746	1.04	0.5	0.32	-0.422
31	CMC	4	-0.186	-0.021	1.15	3.2	0.16	-0.39
32	CMC	4	-0.270	-0.367	1.08	1.9	0.26	-0.368

Note. WMNSQ =Weighted Mean Square; DIF = differential item functioning.
[1] Difference in item difficulty between standard and reduced test in the sample of SEN-L students
[2] Difference in item difficulty between students with SEN-L and general education students.

Table A2
Results of scaling all ‘non-guessing’ students with special educational needs in learning.

Item	Item Format	Text	Difficulty	WMNSQ	t-value (WMNSQ)	Discrimi nation	DIF SEN-L vs gen-ed[1]
1	MC	1	-1.601	1.02	0.3	0.28	0.284
2	MC	1	-2.527	0.98	-0.1	0.26	0.156
3	MC	1	0.542	1.01	0.1	0.41	0.404
4	MA	1	0.413	0.97	-0.7	0.46	1.114
5	MA	1	1.061	0.90	-1.7	0.54	1.216
6	MA	1	1.464	0.98	-0.3	0.41	1.172
7	MA	1	1.504	1.00	0.1	0.35	0.114
8	CMC	1	-2.037	0.98	-0.2	0.30	-0.214
9	CMC	1	-1.332	1.02	0.3	0.31	-0.412
10	MC	2	0.129	0.96	-1.1	0.47	0.63
11	MC	2	0.888	1.01	0.1	0.36	-0.272
12	MC	2	-0.073	0.96	-1.1	0.46	0.502
13	MC	2	0.702	1.10	2	0.28	1.31
14	MC	2	1.519	1.06	0.6	0.26	-0.882
15	MC	3	0.067	1.05	1.2	0.36	0.53
16	MC	3	-0.233	0.91	-2.3	0.53	1.06
17	CMC	3	-1.812	0.92	-0.8	0.42	0.074
18	CMC	3	-2.246	0.94	-0.5	0.32	-0.452
19	CMC	3	-1.07	0.92	-1.4	0.46	-0.19
20	CMC	3	-1.484	0.94	-0.7	0.39	-0.646
21	MC	3	-0.43	1.00	0	0.41	0.268
22	MC	3	-0.438	0.99	-0.1	0.42	0.486
23	MC	3	-0.712	1.04	0.9	0.33	-1.1
24	MC	3	0.532	1.07	1.4	0.30	-0.494
25	MC	4	-1.028	1.08	1.3	0.24	-0.594
26	MC	4	0.326	1.07	1.3	0.33	-0.148
27	MC	4	0.614	1.12	1.3	0.21	-0.298
28	MC	4	-0.067	1.04	0.8	0.34	-0.246
29	MC	4	-0.696	1.03	0.6	0.33	-0.518
30	MC	4	0.833	0.97	-0.3	0.40	-0.3

Item	Item Format	Text	Difficulty	WMNSQ	t-value (WMNSQ)	Discrimi nation	DIF SEN-L vs gen-ed[1]
31	CMC	4	0.112	1.12	2.4	0.22	-0.22
32	CMC	4	-0.301	1.07	1.5	0.30	-0.27

Note. WMNSQ =Weighted Mean Square; DIF = differential item functioning.
[1] Difference in item difficulty between students with SEN-L and general education students.

Table A3

Results of scaling all students with special educational needs in learning of the first missing class.

Item	Item Format	Text	Difficulty	WMNSQ	t-value (WMNSQ)	Discrimi nation	DIF SEN-L vs gen-ed[1]
1	MC	1	-1.583	1.09	0.8	0.26	0.4
2	MC	1	-2.108	0.95	-0.3	0.38	0.688
3	MC	1	0.283	1.01	0.2	0.45	0.23
4	MA	1	0.285	0.97	-0.5	0.45	1.088
5	MA	1	0.99	0.85	-2	0.59	1.244
6	MA	1	1.447	0.99	-0.1	0.38	1.254
7	MA	1	1.485	1.01	0.1	0.33	0.194
8	CMC	1	-2.285	0.93	-0.4	0.37	-0.358
9	CMC	1	-1.459	0.96	-0.4	0.4	-0.438
10	MC	2	-0.013	0.91	-1.8	0.54	0.576
11	MC	2	0.857	0.99	-0.2	0.42	-0.214
12	MC	2	-0.377	0.99	-0.2	0.46	0.28
13	MC	2	0.937	1.13	1.7	0.27	1.646
14	MC	2	1.492	1.13	1	0.14	-0.824
15	MC	3	0.046	1.02	0.4	0.41	0.604
16	MC	3	-0.213	0.93	-1.3	0.57	1.178
17	CMC	3	-1.912	0.9	-0.7	0.51	0.078
18	CMC	3	-2.153	0.94	-0.3	0.34	-0.246
19	CMC	3	-1.059	0.89	-1.3	0.51	-0.074
20	CMC	3	-2	0.96	-0.2	0.35	-1.064
21	MC	3	-0.51	0.98	-0.2	0.42	0.282
22	MC	3	-0.404	0.98	-0.3	0.44	0.616
23	MC	3	-0.94	1.05	0.7	0.3	-1.24
24	MC	3	0.477	1.09	1.3	0.31	-0.458
25	MC	4	-0.906	1.02	0.2	0.39	-0.376
26	MC	4	-0.019	1.05	0.7	0.38	-0.418
27	MC	4	0.393	1.06	0.5	0.28	-0.44
28	MC	4	-0.003	1.11	1.2	0.3	-0.098
29	MC	4	-0.895	1.03	0.4	0.35	-0.642
30	MC	4	0.807	1.32	1.7	-0.19	-0.262

Item	Item Format	Text	Difficulty	WMNSQ	t-value (WMNSQ)	Discrimi nation	DIF SEN-L vs gen-ed[1]
31	CMC	4	-0.07	1.3	3	-0.12	-0.336
32	CMC	4	-0.27	1.11	1.2	0.19	-0.16

Note. WMNSQ =Weighted Mean Square; DIF = differential item functioning.
[2] Difference in item difficulty between students with SEN-L and general education students.

Table A4
Results of scaling all students with special educational needs in learning of the second missing class.

Item	Item Format	Text	Difficulty	WMNSQ	t-value (WMNSQ)	Discrimi nation	DIF SEN-L vs gen-ed[2]
1	MC	1	-1.29	0.98	-0.2	0.36	0.446
2	MC	1	-2.222	0.99	0	0.29	0.302
3	MC	1	0.885	1.05	0.6	0.29	0.598
4	MA	1	0.617	0.98	-0.4	0.41	1.156
5	MA	1	1.295	0.93	-0.7	0.48	1.288
6	MA	1	1.536	0.98	-0.2	0.38	1.074
7	MA	1	1.539	1.01	0.1	0.31	-0.026
8	CMC	1	-1.462	1.01	0.1	0.29	0.21
9	CMC	1	-0.954	1.05	0.7	0.27	-0.19
10	MC	2	0.407	1	0	0.34	0.764
11	MC	2	0.988	1.05	0.6	0.29	-0.324
12	MC	2	0.349	0.96	-0.7	0.43	0.784
13	MC	2	0.441	1.05	1	0.29	0.882
14	MC	2	1.29	1	0.1	0.35	-1.304
15	MC	3	0.307	1.01	0.1	0.36	0.624
16	MC	3	-0.036	0.88	-2.4	0.54	1.112
17	CMC	3	-1.373	0.94	-0.6	0.42	0.36
18	CMC	3	-1.885	0.94	-0.4	0.37	-0.248
19	CMC	3	-0.863	0.95	-0.8	0.45	-0.138
20	CMC	3	-1.017	0.96	-0.5	0.41	-0.328
21	MC	3	-0.146	0.97	-0.7	0.42	0.408
22	MC	3	-0.337	0.98	-0.4	0.39	0.438
23	MC	3	-0.354	0.99	-0.1	0.37	-0.882
24	MC	3	0.621	1.06	1	0.25	-0.554
25	MC	4	-0.84	1.07	1	0.2	-0.55
26	MC	4	0.678	1.03	0.4	0.32	0.076
27	MC	4	0.802	1.18	1.5	0.11	-0.25
28	MC	4	0.092	0.99	-0.2	0.38	-0.218
29	MC	4	-0.504	1.02	0.4	0.31	-0.46
30	MC	4	0.747	0.83	-1.6	0.66	-0.526

Item	Item Format	Text	Difficulty	WMNSQ	t-value (WMNSQ)	Discrimi nation	DIF SEN-L vs gen-ed[2]
31	CMC	4	0.071	1.09	1.8	0.24	-0.402
32	CMC	4	-0.325	1.05	1.1	0.28	-0.434

Note. WMNSQ =Weighted Mean Square; DIF = differential item functioning.
[1] Difference in item difficulty between students with SEN-L and general education students.
